# 2,2-Dimethyl-5-[(3-nitro­anilino)methyl­ene]-1,3-dioxane-4,6-dione

**DOI:** 10.1107/S1600536809031535

**Published:** 2009-08-15

**Authors:** Meng Zhou, Rui Li, Zhen-Yu Ding

**Affiliations:** a State Key Laboratory of Biotherapy, West China Hospital, Sichuan University, Chengdu 610041, People’s Republic of China

## Abstract

The benzene ring of the title compound, C_13_H_12_N_2_O_6_, is twisted away from the planes of the amino­methyl­ene unit and the dioxane ring by 30.13 (4) and 35.89 (4)°, respectively. The dioxane ring exhibits a half-boat conformation, in which the C atom between the dioxane O atoms is 0.553 (8) Å out-of-plane. An intra­molecular N—H⋯O hydrogen bond stabilizes the conformation of the dioxane ring with the amino­methyl­ene group [the dihedral angle between the mean planes of the dioxane ring and the amino­methyl­ene group is 11.61 (4)°].  In the crystal, a three-dimensional framework is built *via* weak inter­molecular N—H⋯O and C—H⋯O inter­actions.

## Related literature

For the synthesis of related compounds, see: Cassis *et al.* (1985 ▶). For the synthesis of related anti­tumor precursors, see Ruchelman *et al.* (2003 ▶). For the crystal structures of related 5-aryl­amino­methyl­ene-2,2-dimethyl-1,3-dioxane-4,6-dione deriv­atives, see Li *et al.* (2009*a* ▶,*b* ▶); Li, Shi *et al.* (2009 ▶).
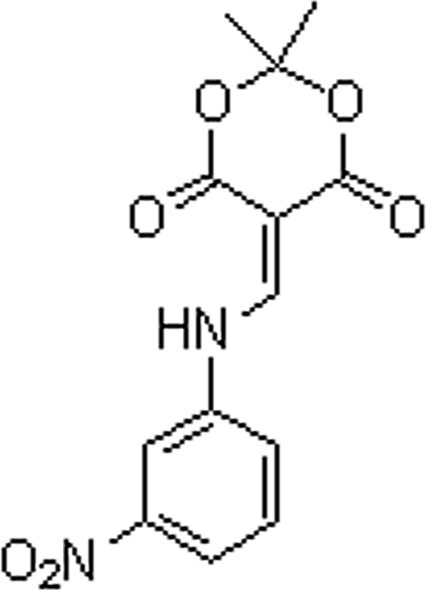


## Experimental

### 

#### Crystal data

C_13_H_12_N_2_O_6_
*M* *_r_* = 292.25
Monoclinic, 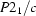

*a* = 11.7900 (13) Å
*b* = 8.7699 (9) Å
*c* = 14.0614 (15) Å
β = 113.8640 (10)°
*V* = 1329.6 (2) Å^3^
*Z* = 4
Mo *K*α radiation
μ = 0.12 mm^−1^
*T* = 153 K
0.20 × 0.10 × 0.10 mm

#### Data collection

Bruker SMART CCD area-detector diffractometer
Absorption correction: none
8116 measured reflections
3052 independent reflections
2572 reflections with *I* > 2σ(*I*)
*R*_int_ = 0.014

#### Refinement

*R*[*F* ^2^ > 2σ(*F* ^2^)] = 0.036
*wR*(*F* ^2^) = 0.101
*S* = 1.05
3052 reflections
197 parameters
H atoms treated by a mixture of independent and constrained refinement
Δρ_max_ = 0.22 e Å^−3^
Δρ_min_ = −0.18 e Å^−3^

### 

Data collection: *SMART* (Bruker, 2001 ▶); cell refinement: *SAINT* (Bruker, 2000 ▶); data reduction: *SAINT*; program(s) used to solve structure: *SHELXS97* (Sheldrick, 2008 ▶); program(s) used to refine structure: *SHELXL97* (Sheldrick, 2008 ▶); molecular graphics: *ORTEP-3* (Farrugia, 1997 ▶); software used to prepare material for publication: *SHELXL97* and *PLATON* (Spek, 2009 ▶).

## Supplementary Material

Crystal structure: contains datablocks I, global. DOI: 10.1107/S1600536809031535/zq2002sup1.cif

Structure factors: contains datablocks I. DOI: 10.1107/S1600536809031535/zq2002Isup2.hkl

Additional supplementary materials:  crystallographic information; 3D view; checkCIF report

## Figures and Tables

**Table 1:** Hydrogen-bond geometry (Å, °)

*D*—H⋯*A*	*D*—H	H⋯*A*	*D*⋯*A*	*D*—H⋯*A*
N1—H1⋯O3	0.882 (16)	2.150 (15)	2.7705 (14)	126.8 (13)
N1—H1⋯O3^i^	0.882 (16)	2.308 (16)	3.1101 (14)	151.2 (13)
C7—H7⋯O4^ii^	0.93	2.58	3.4527 (18)	156
C11—H11⋯O4^iii^	0.93	2.39	3.242 (2)	152
C13—H13⋯O4^ii^	0.93	2.36	3.205 (2)	151

Symmetry codes: (i) 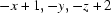; (ii) 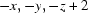; (iii) 
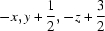
.

**Fig. 1.**
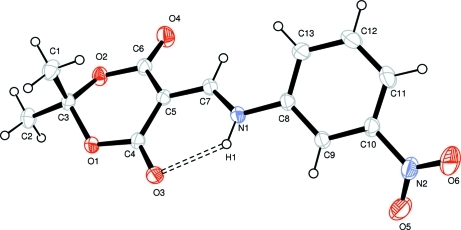
The molecular structure of the title compound, with displacement ellipsoids drawn at the 30% probability level. The intramolecular hydrogen bond is shown as a dashed line.

**Fig. 2.**
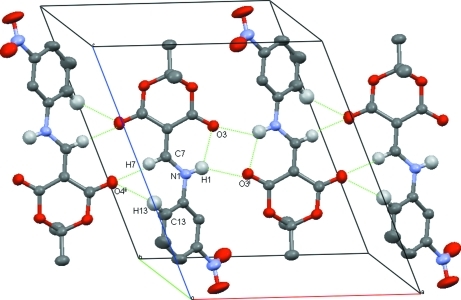
Crystal packing of the title compound, showing the intermolecular hydrogen bonds as dashed lines. [Symmetry codes: (i) -x + 1, -y, -z + 2; (ii) -x, -y, -z + 2].

## supplementary crystallographic information

### Comment

The 4(1*H*)quinolone structure have long attracted pharmacological interest as anticancer agents, anti-malarial agents and reversible (H+/K+) ATPase inhibitors (Ruchelman *et al.*, 2003). 5-Arylaminomethylene-2,2-dimethyl-1,3-dioxane-4,6-diones are the key intermediates which can be used to synthesize the 4(1*H*)quinolone derivatives by thermolysis (Cassis *et al.*, 1985).

The molecular structure of the title compound is shown in Fig. 1. The dihedral angle between the mean planes formed by the benzyl and aminomethylene units is 30.13 (4)°, while the angle between the mean planes of the dioxane ring and the aminomethylene group is only 11.61 (4)° due to the intramolecular N1—H1···O3 hydrogen bond (Table 1). Apart from that, the dioxane ring exhibits a half-boat conformation, in which the C atom between the dioxane oxygen atoms is 0.553 (8) Å out-of-plane.

The three-dimensional framework is built by the weak intermolecular N1—H1···O3^i^, C7—H7···O4^ii^, C13—H13···O4^ii^ and C11—H11···O4^iii^ interactions (Fig. 2).

### Experimental

An ethanol solution (50 ml) of 2,2-dimethyl-1,3-dioxane-4,6-dione (1.44 g, 0.01 mol) and methylorthoformate (1.27 g, 0.012 mol) was heated to reflux for 1 h, then the 3-nitrobenzenamine (1.38 g, 0.01 mol) was added into the solution. The mixture was heated under reflux for another 8 h and then filtered. Single crystals were obtained from the filtrate after 3 days.

### Refinement

The imino H atom was located in a difference Fourier map and refined isotropically. The other H atoms were positioned geometrically and refined using a riding model, with C—H = 0.93 Å and *U*_iso_(H) = 1.2*U*_eq_(C) for aromatic, C—H = 0.96 Å and *U*_iso_(H) = 1.5*U*_eq_(C) for methyl H atoms.

### Crystal data

**Table:**

C_13_H_12_N_2_O_6_	*F*(000) = 608
*M**_r_* = 292.25	*D*_x_ = 1.460 Mg m^−^^3^
Monoclinic, *P*2_1_/*c*	Mo *K*α radiation, λ = 0.71073 Å
*a* = 11.7900 (13) Å	Cell parameters from 4305 reflections
*b* = 8.7699 (9) Å	θ = 2.3–27.6°
*c* = 14.0614 (15) Å	µ = 0.12 mm^−^^1^
β = 113.864 (1)°	*T* = 153 K
*V* = 1329.6 (2) Å^3^	Block, colourless
*Z* = 4	0.20 × 0.10 × 0.10 mm

### Data collection

**Table:**

Bruker SMART CCD area-detector diffractometer	2572 reflections with *I* > 2σ(*I*)
Radiation source: sealed tube	*R*_int_ = 0.014
graphite	θ_max_ = 27.6°, θ_min_ = 2.8°
φ and ω scans	*h* = −15→14
8116 measured reflections	*k* = −11→10
3052 independent reflections	*l* = −18→18

### Refinement

**Table:**

Refinement on *F*^2^	Secondary atom site location: difference Fourier map
Least-squares matrix: full	Hydrogen site location: mixed
*R*[*F*^2^ > 2σ(*F*^2^)] = 0.036	H atoms treated by a mixture of independent and constrained refinement
*wR*(*F*^2^) = 0.101	*w* = 1/[σ^2^(*F*_o_^2^) + (0.045*P*)^2^ + 0.3157*P*] where *P* = (*F*_o_^2^ + 2*F*_c_^2^)/3
*S* = 1.05	(Δ/σ)_max_ = 0.001
3052 reflections	Δρ_max_ = 0.22 e Å^−^^3^
197 parameters	Δρ_min_ = −0.18 e Å^−^^3^
0 restraints	Extinction correction: *SHELXL97* (Sheldrick, 2008), Fc^*^=kFc[1+0.001xFc^2^λ^3^/sin(2θ)]^-1/4^
Primary atom site location: structure-invariant direct methods	Extinction coefficient: 0.0195 (18)

### Special details

**Table:**

Geometry. All e.s.d.'s (except the e.s.d. in the dihedral angle between two l.s. planes) are estimated using the full covariance matrix. The cell e.s.d.'s are taken into account individually in the estimation of e.s.d.'s in distances, angles and torsion angles; correlations between e.s.d.'s in cell parameters are only used when they are defined by crystal symmetry. An approximate (isotropic) treatment of cell e.s.d.'s is used for estimating e.s.d.'s involving l.s. planes.
Refinement. Refinement of *F*^2^ against ALL reflections. The weighted *R*-factor *wR* and goodness of fit *S* are based on *F*^2^, conventional *R*-factors *R* are based on *F*, with *F* set to zero for negative *F*^2^. The threshold expression of *F*^2^ > σ(*F*^2^) is used only for calculating *R*-factors(gt) *etc*. and is not relevant to the choice of reflections for refinement. *R*-factors based on *F*^2^ are statistically about twice as large as those based on *F*, and *R*- factors based on ALL data will be even larger.

### Fractional atomic coordinates and isotropic or equivalent isotropic displacement parameters (Å^2^)

**Table:**

	*x*	*y*	*z*	*U*_iso_*/*U*_eq_	
O1	0.45433 (8)	−0.09916 (10)	1.23372 (6)	0.0370 (2)	
O3	0.47419 (8)	−0.05487 (12)	1.08661 (7)	0.0440 (2)	
O2	0.25826 (8)	−0.13984 (11)	1.23681 (6)	0.0401 (2)	
C4	0.40464 (11)	−0.06725 (13)	1.13054 (9)	0.0322 (2)	
N1	0.26298 (10)	0.01843 (13)	0.91248 (8)	0.0359 (2)	
C8	0.19585 (11)	0.06369 (14)	0.80763 (9)	0.0342 (3)	
C9	0.24224 (11)	0.02544 (14)	0.73506 (9)	0.0347 (3)	
H9	0.3148	−0.0312	0.7538	0.042*	
C3	0.37852 (11)	−0.07324 (14)	1.29110 (9)	0.0337 (3)	
O4	0.08565 (9)	−0.12773 (15)	1.09442 (8)	0.0584 (3)	
C7	0.21114 (11)	−0.01291 (14)	0.97781 (9)	0.0350 (3)	
H7	0.1252	−0.0055	0.9519	0.042*	
O5	0.30012 (13)	−0.07262 (15)	0.57581 (10)	0.0684 (3)	
C6	0.19637 (11)	−0.10611 (15)	1.13438 (9)	0.0381 (3)	
C5	0.27159 (11)	−0.05534 (14)	1.08029 (9)	0.0333 (3)	
C10	0.17796 (12)	0.07365 (16)	0.63388 (9)	0.0403 (3)	
C13	0.08587 (14)	0.14495 (19)	0.77793 (11)	0.0510 (4)	
H13	0.0545	0.1705	0.8268	0.061*	
C2	0.43836 (14)	−0.16096 (17)	1.39116 (10)	0.0459 (3)	
H2A	0.3876	−0.1539	1.4299	0.069*	
H2B	0.5188	−0.1189	1.4315	0.069*	
H2C	0.4468	−0.2660	1.3759	0.069*	
N2	0.22862 (12)	0.03454 (17)	0.55739 (9)	0.0535 (3)	
C1	0.36712 (15)	0.09463 (15)	1.30692 (11)	0.0481 (3)	
H1A	0.3285	0.1440	1.2406	0.072*	
H1B	0.4481	0.1372	1.3444	0.072*	
H1C	0.3174	0.1099	1.3459	0.072*	
C11	0.06978 (15)	0.1552 (2)	0.60191 (11)	0.0586 (4)	
H11	0.0293	0.1874	0.5334	0.070*	
O6	0.19778 (16)	0.1124 (2)	0.47934 (10)	0.0992 (6)	
C12	0.02320 (15)	0.1877 (2)	0.67512 (13)	0.0672 (5)	
H12	−0.0518	0.2393	0.6550	0.081*	
H1	0.3441 (15)	0.0100 (18)	0.9339 (12)	0.051 (4)*	

### Atomic displacement parameters (Å^2^)

**Table:**

	*U*^11^	*U*^22^	*U*^33^	*U*^12^	*U*^13^	*U*^23^
O1	0.0341 (4)	0.0496 (5)	0.0266 (4)	0.0047 (4)	0.0115 (3)	0.0028 (3)
O3	0.0338 (5)	0.0665 (6)	0.0354 (5)	0.0021 (4)	0.0177 (4)	0.0058 (4)
O2	0.0413 (5)	0.0500 (5)	0.0306 (4)	−0.0094 (4)	0.0164 (4)	0.0009 (4)
C4	0.0350 (6)	0.0351 (6)	0.0270 (5)	0.0005 (4)	0.0130 (5)	0.0007 (4)
N1	0.0313 (5)	0.0478 (6)	0.0274 (5)	0.0026 (4)	0.0106 (4)	0.0029 (4)
C8	0.0332 (6)	0.0396 (6)	0.0279 (5)	−0.0008 (5)	0.0105 (5)	0.0033 (5)
C9	0.0328 (6)	0.0388 (6)	0.0302 (6)	0.0001 (5)	0.0106 (5)	0.0030 (5)
C3	0.0375 (6)	0.0384 (6)	0.0263 (5)	−0.0019 (5)	0.0140 (5)	−0.0011 (4)
O4	0.0353 (5)	0.0966 (9)	0.0440 (6)	−0.0133 (5)	0.0167 (4)	0.0000 (5)
C7	0.0312 (6)	0.0427 (6)	0.0295 (6)	−0.0006 (5)	0.0108 (5)	−0.0006 (5)
O5	0.0855 (9)	0.0756 (8)	0.0587 (7)	0.0112 (7)	0.0443 (6)	−0.0031 (6)
C6	0.0352 (6)	0.0482 (7)	0.0324 (6)	−0.0042 (5)	0.0152 (5)	−0.0021 (5)
C5	0.0315 (6)	0.0409 (6)	0.0283 (5)	−0.0007 (5)	0.0128 (5)	−0.0004 (5)
C10	0.0420 (7)	0.0496 (7)	0.0294 (6)	−0.0030 (5)	0.0146 (5)	0.0032 (5)
C13	0.0479 (8)	0.0687 (10)	0.0417 (7)	0.0176 (7)	0.0235 (6)	0.0123 (7)
C2	0.0555 (8)	0.0499 (8)	0.0300 (6)	−0.0003 (6)	0.0147 (6)	0.0052 (5)
N2	0.0583 (8)	0.0716 (9)	0.0339 (6)	−0.0037 (7)	0.0218 (5)	0.0014 (6)
C1	0.0629 (9)	0.0392 (7)	0.0427 (7)	0.0011 (6)	0.0219 (7)	−0.0044 (6)
C11	0.0521 (9)	0.0833 (12)	0.0349 (7)	0.0148 (8)	0.0118 (6)	0.0209 (7)
O6	0.1211 (13)	0.1419 (14)	0.0516 (7)	0.0380 (11)	0.0524 (8)	0.0415 (8)
C12	0.0509 (9)	0.0965 (13)	0.0529 (9)	0.0351 (9)	0.0197 (7)	0.0271 (9)

### Geometric parameters (Å, °)

**Table:**

O1—C4	1.3560 (14)	C7—H7	0.9300
O1—C3	1.4441 (14)	O5—N2	1.2183 (18)
O3—C4	1.2147 (14)	C6—C5	1.4524 (16)
O2—C6	1.3580 (15)	C10—C11	1.370 (2)
O2—C3	1.4348 (15)	C10—N2	1.4665 (17)
C4—C5	1.4401 (17)	C13—C12	1.383 (2)
N1—C7	1.3219 (15)	C13—H13	0.9300
N1—C8	1.4190 (15)	C2—H2A	0.9600
N1—H1	0.882 (16)	C2—H2B	0.9600
C8—C9	1.3800 (17)	C2—H2C	0.9600
C8—C13	1.3875 (18)	N2—O6	1.2164 (17)
C9—C10	1.3802 (17)	C1—H1A	0.9600
C9—H9	0.9300	C1—H1B	0.9600
C3—C1	1.5030 (18)	C1—H1C	0.9600
C3—C2	1.5053 (17)	C11—C12	1.378 (2)
O4—C6	1.2092 (16)	C11—H11	0.9300
C7—C5	1.3754 (16)	C12—H12	0.9300
			
C4—O1—C3	117.92 (9)	C4—C5—C6	119.70 (10)
C6—O2—C3	117.84 (9)	C11—C10—C9	123.24 (12)
O3—C4—O1	118.31 (11)	C11—C10—N2	118.87 (12)
O3—C4—C5	124.71 (11)	C9—C10—N2	117.88 (12)
O1—C4—C5	116.96 (10)	C12—C13—C8	119.52 (13)
C7—N1—C8	124.04 (10)	C12—C13—H13	120.2
C7—N1—H1	119.2 (10)	C8—C13—H13	120.2
C8—N1—H1	116.7 (10)	C3—C2—H2A	109.5
C9—C8—C13	120.26 (11)	C3—C2—H2B	109.5
C9—C8—N1	118.62 (11)	H2A—C2—H2B	109.5
C13—C8—N1	121.12 (11)	C3—C2—H2C	109.5
C8—C9—C10	118.11 (11)	H2A—C2—H2C	109.5
C8—C9—H9	120.9	H2B—C2—H2C	109.5
C10—C9—H9	120.9	O6—N2—O5	123.59 (14)
O2—C3—O1	109.91 (9)	O6—N2—C10	117.94 (14)
O2—C3—C1	110.44 (11)	O5—N2—C10	118.46 (12)
O1—C3—C1	110.45 (10)	C3—C1—H1A	109.5
O2—C3—C2	106.13 (10)	C3—C1—H1B	109.5
O1—C3—C2	106.25 (10)	H1A—C1—H1B	109.5
C1—C3—C2	113.48 (11)	C3—C1—H1C	109.5
N1—C7—C5	126.51 (11)	H1A—C1—H1C	109.5
N1—C7—H7	116.7	H1B—C1—H1C	109.5
C5—C7—H7	116.7	C10—C11—C12	117.54 (13)
O4—C6—O2	118.33 (11)	C10—C11—H11	121.2
O4—C6—C5	125.30 (12)	C12—C11—H11	121.2
O2—C6—C5	116.25 (10)	C11—C12—C13	121.26 (14)
C7—C5—C4	122.25 (10)	C11—C12—H12	119.4
C7—C5—C6	117.66 (11)	C13—C12—H12	119.4
			
C3—O1—C4—O3	163.48 (11)	O3—C4—C5—C6	165.88 (12)
C3—O1—C4—C5	−18.46 (15)	O1—C4—C5—C6	−12.04 (17)
C7—N1—C8—C9	−149.73 (12)	O4—C6—C5—C7	7.2 (2)
C7—N1—C8—C13	30.4 (2)	O2—C6—C5—C7	−176.78 (11)
C13—C8—C9—C10	1.64 (19)	O4—C6—C5—C4	−165.75 (14)
N1—C8—C9—C10	−178.26 (11)	O2—C6—C5—C4	10.23 (18)
C6—O2—C3—O1	−50.49 (14)	C8—C9—C10—C11	−1.1 (2)
C6—O2—C3—C1	71.61 (13)	C8—C9—C10—N2	179.15 (11)
C6—O2—C3—C2	−164.99 (10)	C9—C8—C13—C12	−0.1 (2)
C4—O1—C3—O2	48.46 (13)	N1—C8—C13—C12	179.74 (15)
C4—O1—C3—C1	−73.64 (14)	C11—C10—N2—O6	22.6 (2)
C4—O1—C3—C2	162.88 (10)	C9—C10—N2—O6	−157.58 (15)
C8—N1—C7—C5	−179.14 (12)	C11—C10—N2—O5	−158.17 (16)
C3—O2—C6—O4	−161.51 (12)	C9—C10—N2—O5	21.6 (2)
C3—O2—C6—C5	22.23 (16)	C9—C10—C11—C12	−1.0 (3)
N1—C7—C5—C4	1.6 (2)	N2—C10—C11—C12	178.78 (16)
N1—C7—C5—C6	−171.23 (12)	C10—C11—C12—C13	2.5 (3)
O3—C4—C5—C7	−6.8 (2)	C8—C13—C12—C11	−2.0 (3)
O1—C4—C5—C7	175.30 (11)		

### Hydrogen-bond geometry (Å, °)

**Table:**

*D*—H···*A*	*D*—H	H···*A*	*D*···*A*	*D*—H···*A*
N1—H1···O3	0.882 (16)	2.150 (15)	2.7705 (14)	126.8 (13)
N1—H1···O3^i^	0.882 (16)	2.308 (16)	3.1101 (14)	151.2 (13)
C7—H7···O4	0.93	2.48	2.8026 (18)	101
C7—H7···O4^ii^	0.93	2.58	3.4527 (18)	156
C11—H11···O4^iii^	0.93	2.39	3.242 (2)	152
C13—H13···O4^ii^	0.93	2.36	3.205 (2)	151

Symmetry codes: (i) −*x*+1, −*y*, −*z*+2; (ii) −*x*, −*y*, −*z*+2; (iii) −*x*, *y*+1/2, −*z*+3/2.
